# SKELETAL MUSCLE ENERGETICS ARE ASSOCIATED WITH PERFORMANCE FATIGABILITY

**DOI:** 10.1093/geroni/igad104.2083

**Published:** 2023-12-21

**Authors:** Yujia (Susanna) Qiao, Adam Santanasto, Peggy Cawthon, Daniel Forman, Stephen Kritchevsky, Barb Nicklas, Anne Newman, Nancy Glynn

**Affiliations:** University of Pittsburgh, Pittsburgh, Pennsylvania, United States; University of Pittsburgh, Pittsburgh, Pennsylvania, United States; California Pacific Medical Center, Research Institute, San Francisco, California, United States; University of Pittsburgh, Pittsburgh, Pennsylvania, United States; Wake Forest School of Medicine, Winston Salem, North Carolina, United States; Wake Forest School of Medicine, Winston Salem, North Carolina, United States; University, Pittburgh, Pennsylvania, United States; University of Pittsburgh School of Public Health, Pittsburgh, Pennsylvania, United States

## Abstract

Performance fatigability manifests as insufficient energy to complete daily physical tasks and worsens with aging, exacerbating vulnerability to disability. Skeletal muscle energetics also declines with aging. Thus, we hypothesized muscle energetics may be an important contributor to performance fatigability. In the Study of Muscle, Mobility and Aging (SOMMA), participants completed a usual-paced 400m walk while wearing a wrist-worn ActiGraph, from which raw data were used to derive the Pittsburgh Performance Fatigability Index (PPFI, higher=more severe fatigability) that quantifies percent decline in the entire individual cadence-versus-time trajectory. Maximal oxidative phosphorylation (maxOXPHOS) in skeletal muscle mitochondria was quantified in vitro using high-resolution respirometry in permeabilized fiber bundles from vastus lateralis muscle biopsies. Maximal adenosine triphosphate production (ATPmax) was assessed in vivo by 31P magnetic resonance spectroscopy. We conducted separate tobit regressions to examine associations of maxOXPHOS and ATPmax with PPFI, adjusting for technician/site, age, sex, race, height, weight, and mins/day moderate-to-vigorous physical activity measured by ActiGraph in free-living, in N=795 participants with complete PPFI scores and >1 energetics measure (70-94 yrs, 58% women). Median PPFI scores were 1.4% [IQR: 0-2.9%]. After adjustment, each SD (18.4 pmol/(s*mg)) lower maxOXPHOS was associated with 0.55% (95% CI: 0.26, 0.85) higher PPFI scores, while each SD (0.2 mM/sec) lower ATPmax was associated with 0.54% (95% CI: 0.27, 0.81) higher PPFI scores. Our results indicate that lower skeletal muscle energetics were associated with more severe performance fatigability. This suggests that therapeutics targeting muscle energetics may thereby potentially mitigate fatigability and lessen susceptibility to disability among older adults.

